# Role of indocyanine green in anomalous arterial supply to the normal dorsobasal segment of the lung

**DOI:** 10.1186/s13019-022-01791-0

**Published:** 2022-03-23

**Authors:** Yoshihito Iijima, Masahito Ishikawa, Shun Iwai, Nozomu Motono, Katsuo Usuda, Masafumi Morinaga, Shigeki Yamagishi, Kiyoshi Koizumi, Hidetaka Uramoto

**Affiliations:** 1 Department of Thoracic Surgery, Kanazawa Medical University, 1-1 Daigaku, Uchinada-machi, Kahoku-gun, Ishikawa, 920-0293 Japan; 2 Department of Palliative Care, Miyuki Hospital, Kumamoto, Japan; 3 Department of Thoracic Surgery, Aidu Chuo Hospital, Fukushima, Japan

**Keywords:** Anomalous systemic arterial supply, Indocyanine green, Lung resection, Video-assisted thoracoscopic surgery

## Abstract

**Background:**

Anomalous systemic arterial supply to normal basal lung segments is a rare congenital malformation, in which aberrant arteries arising from the systemic circulation flow into the basal segment of the lung and return to normal pulmonary veins without abnormal bronchial branching. It presents a left-to-right shunt, resulting in volume overload of the pulmonary circulation, and consequently, pulmonary hypertension. Therefore, nearly all cases require surgery. Herein, we present a case, in which indocyanine green was used to demarcate the lung segment perfused by an anomalous systemic artery.

**Case presentation:**

A 15-year-old boy was diagnosed with an anomalous artery originating from the celiac artery and supplying the right dorsobasal lung segment (S^10^). Via three-port video-assisted thoracoscopic surgery, the anomalous artery was ligated and processed with an auto-stapler. Indocyanine green was injected intravenously to identify the tissue perfused by the anomalous artery, and the lung was resected.

**Conclusions:**

With anomalous systemic arterial supply to normal basal lung segments, indocyanine green can be particularly helpful in identifying the boundaries of the perfused area. Then, the affected tissue can be resected by thoracoscopic surgery.

**Supplementary Information:**

The online version contains supplementary material available at 10.1186/s13019-022-01791-0.

## Background

Anomalous systemic arterial supply (ASA) to normal basal lung segments is a rare congenital malformation, in which aberrant arteries from the systemic circulation flow into the basal segments of the lung and return to the normal pulmonary veins, with normal bronchial branching [1]. Although it has been classified as Pryce type I pulmonary sequestration (PS), in recent years, it is considered an independent condition. It presents a left-to-right shunt because blood from the systemic circulation returns to the pulmonary veins, resulting in volume overload of the pulmonary circulation, and consequently, pulmonary hypertension. In advanced cases, hemoptysis and heart failure occur due to pulmonary hypertension; therefore, nearly all cases require surgery. Herein, we report a case, in which an aberrant artery arose from the celiac artery and supplied the right dorsobasal lung segment (S^10^). Indocyanine green (ICG) was particularly useful for identifying the demarcation line of the perfused area.

## Case presentation

**Fig. 1:**
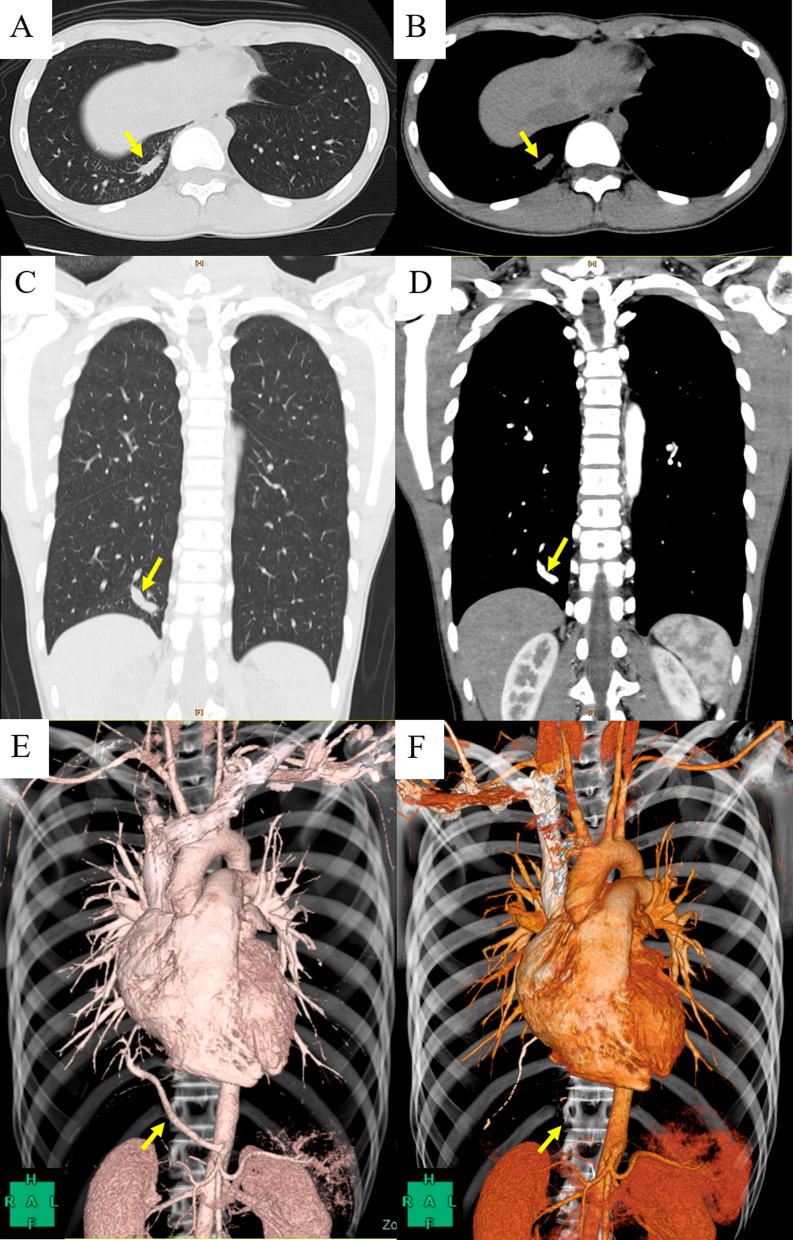
Non-contrasted axial (**a**, **b**) and coronal (**c**) computed tomography (CT) images; **d** contrast-enhanced coronal CT image; and **e** three-dimensional (3D) angiography showed the aberrant artery from the celiac artery (yellow arrow) flowing into the right dorsobasal segment (S10). **f** The findings of 3D angiography at the 3-month follow-examination showed that the remnants of the anomalous artery had become cord-like, without aneurysmal formation

A 15-year-old boy was found to have an abnormal shadow on a chest radiographic examination at a school checkup. He was asymptomatic and had no history of heart disease. Physical examination and laboratory findings were within the normal limits. Chest radiographic findings showed a slight opacity around the right cardiophrenic angle. Non-contrasted chest computed tomography (CT) findings showed a rod-shaped shadow from the inside of the right S^10^ segment toward the pulmonary ligaments (Fig. 1a–c). On contrast-enhanced (CE) chest CT and three-dimensional angiography indicated that aberrant branches from the celiac artery flowed into the right S^10^ segment (Fig. 1d–e). Bronchoscopy was not performed because of declination of consent. Meanwhile, CT findings showed no abnormal bronchial bifurcation. Therefore, we diagnosed ASA to the normal S^10^ segment.

**Fig. 2:**
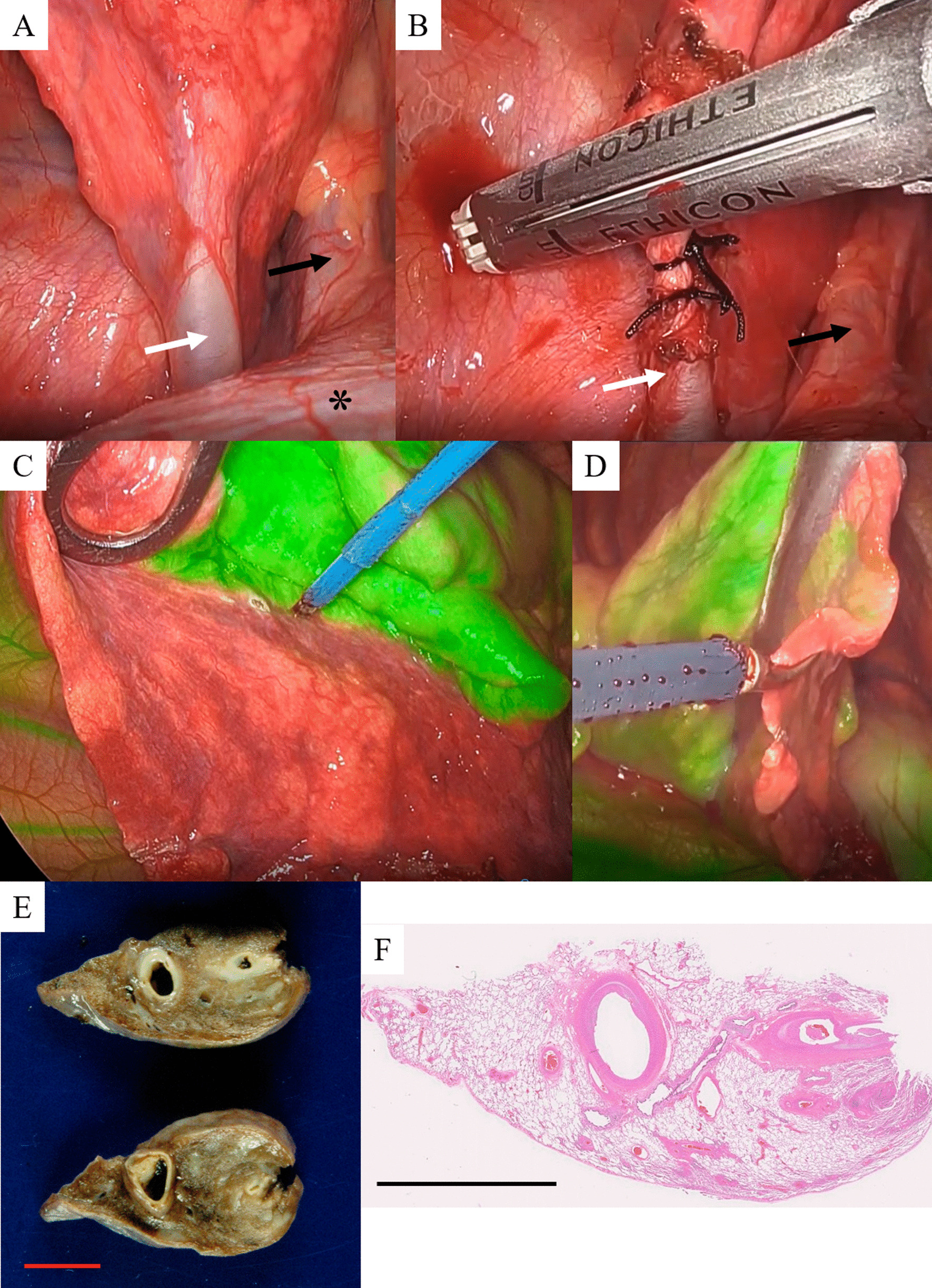
Intraoperative and pathological findings. **a** The aberrant artery (white arrow) was identified. **b** The central side of the aberrant artery (white arrow) was ligated, and the artery was processed with an auto-stapler. The black arrow indicates the inferior vena cava and the asterisk indicates the diaphragm. **c** The region perfused by the anomalous artery was clearly shown by intravenous injection of ICG, and **d** the segment was resected using an auto-stapler. **e** Macroscopically, a large blood vessel dilated in the resected lung was observed. The length of the red bar is 1.0 cm. **f** Histopathologically, the endometrium of the dilated aberrant artery was thickened, and the intime thickening continued to the peripheral pulmonary artery (Hematoxylin–eosin stain, loupe magnification). The length of the black bar is 1.0 cm

Given the option of either catheter embolization of the anomalous artery or lung resection, the patient chose the latter, and we performed three-port video-assisted thoracic surgery as follows. First, the aberrant artery was identified. When the lower lobe of the right lung was raised to the cranial side and the pulmonary ligament was detached, an aberrant artery that ascended through the diaphragm and flowed into the S^10^ segment was confirmed. The central side of the artery was ligated, and the artery was processed with an auto-stapler (Fig. 2a, b). The outer diameter of the aberrant artery was 8 mm. Subsequently, to visualize the demarcation line based on near- infrared (NIR) fluorescence imaging, 5 mg of ICG was injected intravenously to identify the area perfused by the anomalous artery. We used 1688 AIM (Stryker, Tokyo, Japan) NIR thoracoscopy system in this case. Then, the lung parenchyma was resected using an auto-stapler (Fig. 2c, d). Finally, we completed the wedge resection of S^10^ segment. Histopathologically, the endometrium of the dilated aberrant artery was thickened, and the intime thickening continued to the peripheral pulmonary artery (Fig. 2e, f). In addition, plexiform lesions and vasculitis were found in the pulmonary artery. These were considered to be findings of pulmonary hypertension due to ASA. At the 3-month follow up, the patient was in good health and CE-CT showed that the remnants of the anomalous artery had become cord-like, without aneurysmal formation (Fig. 1f). Written informed consent was obtained from the patient for the publication of this report and its accompanying images.

## Discussion and conclusions

ASA to the normal basal segments was formerly known as PS. Most aberrant systemic arteries that drain into the lungs originate directly from either the thoracic or abdominal aorta. Wei et al. examined 2625 cases of PS; only one case originated from the celiac artery, an extremely rare finding [2]. Even in Japan, there were only 10 reported cases in the last 30 years (Table 1) [3, 4]. Seven patients were male and three were female, with an average age of 26.2 years. In seven and three cases, ASA was observed on the right and left sides, respectively. All patients underwent surgery. Until 2010, there were many reported cases, in which the aberrant artery was ligated and dissected; however, recently, with the development of auto-suture, most cases were treated aberrant artery with auto-suture. However, a previous report indicated that it is better to ligate the central side of auto-suture as insurance for the event of misfire [3].

**Table 1:** Japanese cases of interlobar pulmonary sequestration with aberrant artery from the celiac artery in the last 30 years

Case	Sex	Age	Symptom	Perfusion area	Artery number	Artery diameter (mm)	Treatment	Complications	Treatment for abrrant artery	References
1	M	6	Pneumonia, high fiver	Rt S9-S10	1	5	Lob	ND	LD	[3]
2	F	39	–	Rt basal	2	3, 5	Lob	None	LD	[3]
3	M	13	Cough, high fever	Rt S9-S10	1	10	Lob	None	LD	[3]
4	M	23	Cough, high fever	Rt S10	1	ND	Lob	None	LD	[3]
5	F	29	Cough, high fever	Lt lower lobe	1	ND	Lob	None	ND	[3]
6	F	43	–	Lt basal	1	ND	Seg	None	LD	[3]
7	M	26	Hemosputum	Rt basal	1	ND	Lob	None	AS	[3]
8	M	26	–	Lt S10	1	ND	Wed	None	AS	[3]
9	M	42	–	Lt basal	1	33	Lob	None	AS	[4]
10	M	15	–	Rt S10	1	8	Wed	None	AS	Our case

M; male, F; female, Rt; right, Lt; left, ND; not discribed, Lob; lobectomy, Seg; segmentectomy, Wed; wedge resection, LD; ligatet and dissection, AS; auto-stapler

The management of asymptomatic PS is controversial. Surgical resection is recommended because of the possibility of hemorrhage and heart failure secondary to the progression of pulmonary hypertension in advanced cases. In recent years, successful cases of endovascular coil embolization have also been reported [5].

In recent years, ICG has been extensively used in the field of general thoracic and pulmonary surgery, such as in the identification of the demarcation line in lung segmentectomy or evaluation of the blood flow of the bronchial anastomosis in bronchoplasty [6, 7].

The strategy underlying surgery for ASA to the normal basal segment is excision of the lung area perfused by the anomalous artery. We have previously reported cases, in which ICG was useful in identifying the borders of Pryce type III intralobular sequestration [8]. To our knowledge, this is the first study to use ICG to identify the area of the normal basal segment perfused by an anomalous arterial supply (Pryce type I intralobular PS); we could clearly identify the demarcation line of the perfused area with ICG. In conclusion, ICG was particularly helpful in identifying the boundaries of the perfused area, and surgery was performed safely.

## Supplementary Information

**Additional file 1:** Intraoperative findings. The aberrant artery that ascended through the diaphragm and flowed into the S10 segment was confirmed. The central side of the aberrant artery was ligated, and the artery was processed with an auto-stapler. The region perfused by the anomalous artery was clearly shown by intravenous injection of indocyanine green, and the segment was resected using an auto-stapler.

## Data Availability

All data generated or analyzed during this study are included in this published article.

## Abbreviations

ASA: Anomalous systemic arterial supply
PS: Pulmonary sequestration
CT: Computed tomography
CE: Contrast-enhanced
ICG: Indocyanine green
NIR: Near-infrared
